# The transition of late preterm

**DOI:** 10.1186/1824-7288-40-S2-A2

**Published:** 2014-10-09

**Authors:** Maria Pia De Carolis, Carmen Cocca, Serena Antonia Rubortone, Giovanni Pinna, Sara De Carolis, Silvia Salvi, Costantino Romagnoli

**Affiliations:** 1 Division of Neonatology, Department of Obstetrics, Gynecology and Pediatrics, Catholic University of Sacred Heart, Rome, 00168, Italy; 2 Division of Maternal Medicine, Department of Obstetrics, Gynecology and Pediatrics, Catholic University of Sacred Heart, Rome, 00168, Italy

## Background

The transition from the intra- to the extra-uterine life is characterized by major physiological changes in respiratory and hemodynamic functions [1]; moreover, the intrauterine thermostability has to been replaced by the neonatal termoregulation [2]. Many of the antepartum and intrapartum risk factors associated with the need of resuscitation may be present in late-preterm neonates (34^0/7^-36^6/7^ weeks) [3]. It is also reported a double risk of Caesarean Section (CS) in case of late-preterm compared to term deliveries [4]. Our objective was to evaluate the transition period in late-preterm infants in particular considering the need for resuscitation and the incidence of hypothermia.

## Materials and methods

This was a retrospective study of all late preterm neonates during a 1-year period from January 2013. Gestational Age (GA) was calculated as a function of the date of last menstrual period and/or biometrics assigned from the ultrasound measurement of the first trimester. Type of pregnancy (singleton or multiple), use of antepartum steroid therapy, maternal medical disorders, obstetric and/or fetal complications, intrapartum fetal distress, birth weight (BW), gender, Apgar score, need for resuscitation were collected from medical records Rectal temperature was measured in all neonates at birth and at admission to nursery.

## Results

During the study period there were a total of 3354 births. The number of preterm neonates was 478 (14.2%), of these 279 (58%) were late-preterm (249 singleton pregnancy and 30 multiple pregnancy). Three neonates were excluded due to *in utero* fetal death. Table 1 summarizes the characteristics of the population according to GA: 34 weeks of gestation (Group I), 35 weeks (Group II) and 36 weeks (Group III). The twins rate was significantly higher (p<0.001) in Group I than the others. The CS rate was similar among the groups and increased in comparison to that reported in our Department for term deliveries (44%). A higher number of neonates with Apgar score <7^1^ was present in Group I in comparison to the others, as well as a higher number of neonates requiring resuscitation, independently of the mode of delivery. In Group II and III, all neonates requiring resuscitation were born by CS. Higher number of neonates with mild hypothermia at admission was detected in Group I. Considerable variations occur in the temperature values in all infants in DR as well as during the transport to the nursery.

**Table 1:** Neonatal characteristics by gestational age group

Gestational age (weeks^+days^ of gestation)			
	34^+0^-34^+6^	35^+0^-35^+6^	36^+0^-36^+6^

Number (%)	62 (22.5)	77 (27.9)	137(49.6)
Singletons, n (%)	40 (64.5)	65 (84.4)	117 (85.4)
Multiples, n (%)	22 (35.4)	12 (15.6)	20 (14.6)
Vaginal Delivery, n (%)	14 (22.5)	23 (29.8)	51 (37.2)
Caesarean-section, n (%)	48 (77.5)	54 (70.2)	86 (62.8)
1 min Apgar , mean (DS)	7 ± 2	8 ± 1	9 ± 1
1 min Apgar <7, n (%)	15 (24.2)	4 (5.2)	4 (2.9)
5-min Apgar, mean (DS)	9 ± 1	9 ± 1	9 ± 1
5-min Apgar <7, n (%)	2 (3,2%)	2 (2,5%)	0
Positive Pressure Ventilation, n (%)	15	4	4
Endotracheal Intubation, n (%)	6 (9.7)	3 (3.9)	1 (0.7)

## Conclusion

Late-preterm birth by CS is associated with significant GA-dependent neonatal depression. Additional close monitoring and timely intervention are necessary in the management of these infants in DR.
